# Reassessing the Proposed Creatine–PrP Axis in Endometriosis: Methodological and Mechanistic Considerations

**DOI:** 10.1002/advs.202521907

**Published:** 2026-07-06

**Authors:** Marco Machado

**Affiliations:** ^1^ Fundação Universitária de Itaperuna Itaperuna RJ Brazil

**Keywords:** creatine metabolism, endometriosis, ferroptosis resistance, metabolic regulation, prion protein

## Abstract

The research by Chen et al. (2024) presents an innovative hypothesis linking creatine metabolism to ferroptosis resistance in endometriotic stromal cells via PrP suppression. While conceptually valuable, methodological aspects warrant reflection. A primary concern involves the supraphysiological creatine concentrations used, whose translational relevance is questionable without pharmacokinetic data confirming these levels in the peritoneal environment, particularly in light of available clinical metabolomics data suggesting differences in creatine levels in peritoneal fluid under physiological and pathological conditions. Furthermore, the proposed direct suppression of PrP ferrireductase activity would benefit from complementary biochemical validation using purified systems and affinity measurements to consolidate the molecular mechanism. Although human samples were included, the small cohort size and lack of control for confounding variables limit the specificity of interpretation, source attribution, and the external generalizability of the findings. Finally, future studies should target endogenous creatine synthesis or transport to clarify whether creatine is a causal driver of ferroptosis resistance or simply associated with the inflammatory microenvironment. Overall, Chen et al. provide an exciting contribution; these constructive considerations aim to support future refinements that will solidify the proposed model and its clinical significance.

The paper by Chen et al. [1] proposes a novel relationship between creatine metabolism, PrP modulation, and ferroptosis resistance in endometrial stromal cells. This ambitious work offers an original and potentially relevant mechanistic hypothesis for understanding cell survival in endometriosis. However, certain methodological and conceptual aspects warrant careful consideration—not to undermine the authors' proposition, but to contribute to its depth and to strengthen the presented conclusions. The first issue pertains to the concentrations of creatine used in the in vitro and in vivo experiments, particularly at the higher experimental ranges (e.g., 1, 10, and 100 mM), which may exceed commonly reported physiological levels, including under supplementation. Importantly, a clear distinction should be made between endogenous creatine enrichment observed in patient‐derived samples and the pharmacological exogenous concentrations employed in mechanistic experiments, as these represent fundamentally different biological contexts. Clinical metabolomics analyses of peritoneal fluid from patients with and without endometriosis have identified metabolic alterations associated with the disease; however, the specific concentration ranges of creatine in this compartment remain insufficiently characterized across studies, and direct equivalence with experimental exposure conditions cannot be assumed [2, 3]. Furthermore, it should be clarified that creatine concentrations measured in peritoneal fluid and creatine content normalized to endometriotic stromal cells (ESCs) represent distinct types of measurements and, therefore, cannot be directly compared. While such approaches are common in exploratory studies, it would be crucial to clarify the extent to which these concentrations reflect relevant physiological conditions within the peritoneal environment or the microenvironment of endometriotic lesions, as the absence of pharmacokinetic data or direct post‐administration measurements hinders biological extrapolation. Furthermore, although human samples were included, the small cohort size and lack of control for confounding variables limit the specificity of interpretation, source attribution, and the external generalizability of the findings. Another point meriting discussion concerns the proposed mechanism involving the direct binding of creatine to PrP and the subsequent suppression of its ferrireductase activity. Importantly, the original study does not rely solely on proteomic inference, as it also incorporates functional genetic‐perturbation approaches, including PRNP overexpression and silencing experiments, as well as creatine rescue assays, which collectively support a functional involvement of PrP in the observed phenotype. However, while these findings strengthen the biological plausibility of the model, they do not yet establish a direct molecular interaction, which would require independent biochemical validation, including binding affinity measurements and enzymatic activity assays using purified protein. Thus, while the proposed model is plausible and coherent with prior literature on PrP and iron homeostasis, it remains dependent on complementary biochemical evidence to consolidate the suggested causal relationship. Furthermore, the analysis involving human samples represents a strength of the study, as it confers clinical relevance to the investigated phenomenon; nevertheless, the reduced sample size and the absence of systematic control for potential confounding variables, such as diet, supplementation, renal function, or the exact phase of the menstrual cycle, make it challenging to conclude to what extent the observed increase in creatine is a specific marker of endometriosis or a reflection of uncontrolled external variability, suggesting that future studies with enlarged cohorts and more detailed inclusion criteria, including metabolic, dietary, and renal parameters, would be extremely valuable in this regard. An additional point that merits consideration is the partial disconnect between patient‐derived observations and mechanistic experimentation. While the study includes analyses of peritoneal fluid, endometriotic stromal cells (EESCs/NESCs), and tissue expression, several key mechanistic assays were conducted in human endometrial stromal cells (HESCs), which may not fully recapitulate the molecular and metabolic context of patient‐derived endometriotic cells. This distinction introduces an important limitation in establishing direct causal links between clinical observations and experimental mechanisms. Finally, the biological interpretation would benefit from additional approaches testing inverse causality, such as manipulations of endogenous creatine synthesis or its cellular transport, because if creatine indeed plays a determinant role in ferroptosis resistance within endometriotic lesions, a reduction in its cellular availability is expected to modify the observed phenotype, helping to delineate whether creatine acts as a causal agent or merely as an associated component of the disease's inflammatory environment. In summary, the article by Chen et al. represents a stimulating contribution and opens up new avenues of investigation into metabolism and redox biology in endometriosis. The observations above are not intended to question the merit of the work but to offer points that, if explored in subsequent studies, could significantly strengthen the understanding of the proposed mechanism and its potential clinical impact. Taken together, the available data support a plausible involvement of PrP in creatine‐mediated ferroptosis resistance, although key aspects of direct molecular interaction and enzymatic modulation remain to be independently validated. Overall, the article inaugurates a promising line of research, and further discussion of these aspects is expected to contribute to important advancements in the field.

## Author Contributions

Marco Machado is responsible for writing, reviewing, and finalizing the manuscript.

## Funding

The author(s) received no financial support for the research, authorship, and/or publication of this article.

## Ethics Statement

Ethical approval and informed consent were not required for this study, as it did not involve human participants or animal subjects.

## Conflicts of Interest

The authors declare no conflicts of interest.

## Data Availability

No datasets were generated or analyzed during the current study, and therefore, data sharing is not applicable.
